# Relationships Between Body Composition and Performance in the High-Intensity Functional Training Workout “Fran” are Modulated by Competition Class and Percentile Rank

**DOI:** 10.3389/fphys.2022.893771

**Published:** 2022-05-27

**Authors:** Gerald T. Mangine, Jacob M. McDougle, Yuri Feito

**Affiliations:** ^1^ Exercise Science and Sport Management, Kennesaw State University, Kennesaw, GA, United States; ^2^ Kinesiology, University of Connecticut, Storrs, CT, United States; ^3^ American College of Sports Medicine, Indianapolis, IN, United States

**Keywords:** CrossFit^®^, athlete, dual energy X-ray absoptiometry, HIFT, body fat percentage, bone mineral denisty

## Abstract

This study examined relationships between body composition and high-intensity functional training (HIFT) workout performance. Fifty-seven men (31.4 ± 6.9 years, 177.2 ± 7.5 cm, 84.7 ± 8.5 kg) and thirty-eight women (29.2 ± 6.4 years, 166.6 ± 6.1 cm, 66.5 ± 7.7 kg) with HIFT experience (≥6 months) reported completing “Fran” (21-15-9 repetitions of barbell thrusters and pull-ups) in 4.78 ± 2.22 min and 6.05 ± 2.84 min, respectively, and volunteered to complete dual-energy X-ray absorptiometry assessments. Participants were grouped by competition class (men, women, master’s men, master’s women) and percentile rank in “Fran” (≤25th percentile, 25–75th percentiles, ≥75th percentile). Two-way analyses of variance revealed expected differences (*p* < 0.001) between men and women in non-bone lean mass (NBLM), fat-free mass index, and fat mass, and more NBLM (10.6–10.8 kg) and less fat mass (2.7–5.2 kg) in >75th percentile compared to other percentiles. Most body composition measures were significantly (*p* < 0.05) related to performance in men and women but limited in master’s men; no relationships were seen in master’s women. “Fran” time was negatively correlated to NBLM and fat-free mass index in all percentile groups (*ρ* = -0.37 to -0.64) and bone mineral characteristics for >25th percentile (*ρ* = −0.41 to −0.63), and positively correlated to fat mass in 25–75th percentiles (*ρ* = 0.33–0.60). No other relationships were seen in ≤25th percentile. The influence of body composition on “Fran” time appears to vary by both competition class and percentile rank. Though training to increase lean mass always seems relevant, reducing body fat only appears relevant in mid-skilled trainees and when it is outside healthy parameters.

## Introduction

High-intensity functional training (HIFT) variably programs multimodal, functional movements designed to be performed at a relatively high intensity (within the context of prescribed repetitions or durations) in an effort to promote general physical fitness across multiple physiological parameters (Feito et al., 2018b). This method is reflected in the design of HIFT competition workouts, which require aptitude in various combinations of fitness domains (e.g., strength, cardiorespiratory fitness, and sports-specific skill). Indeed, relationships have been observed between most investigated physiological traits and HIFT performance (Bellar et al., 2015; Butcher et al., 2015; Feito et al., 2018a; Dexheimer et al., 2019; Carreker and Grosicki, 2020; Mangine et al., 2020; Zeitz et al., 2020) and it is unclear which is most important. Without clarity, those aiming to train effectively have little choice but to address all relevant areas of fitness. One reason for the lack of clarity is that most studies have limited their examination to only one or a few specific fitness domains (Bellar et al., 2015; Butcher et al., 2015; Feito et al., 2018a; Dexheimer et al., 2019; Carreker and Grosicki, 2020; Zeitz et al., 2020). That is, few comparisons have been made among traits as to their relative importance. To the best of our knowledge, only one investigation attempted to comprehensively determine the relative importance of multiple physiological characteristics (e.g., body composition, muscle morphology, hormonal concentrations, resting metabolism, aerobic capacity, and anaerobic power), in addition to training experience and sport-specific skill (Mangine et al., 2020). Though most variables were related to performance (in six competition workouts), the most consistent predictor involved some measure of body composition (i.e., body fat percentage, body density, or skeletal muscle cross-sectional area). This was an uncommon finding compared to other studies on this topic.

Body composition was not previously considered to be statistically important (Bellar et al., 2015; Butcher et al., 2015; Feito et al., 2018a; Dexheimer et al., 2019; Zeitz et al., 2020), likely because its role in those studies was limited to descriptive purposes. Only three studies have used it as a predictor and in limited capacity (Butcher et al., 2015; Carreker and Grosicki, 2020; Zeitz et al., 2020). Butcher et al. (2015) reported that body mass was related to, but not the best predictor of “Grace” (*r* = −0.67) and the CrossFit® Total (*r* = 0.77). Zeitz et al. (2020) reported that body mass and body fat percentage, measured by bioelectrical impedance analysis, were related (*r* = -0.46 and *r* = 0.53, respectively) to a 15-min circuit of 19 wall balls and 19 calories on a rowing ergometer where the goal was to complete “*as many repetitions as possible*” (AMRAP), but not a modified version of “Fran” that replaced pull-ups with bar-facing burpees. Stronger correlations (*r* ≥ 0.56) were seen from several other performance measures collected in that study, with aerobic capacity being the best predictor (*r* = 0.68) of the 15-min circuit. In fact, the only other study to observe body composition as the best predictor of HIFT used the results of dual-energy X-ray absorptiometry (DXA) in relation to a scaled version of “Murph” (Carreker and Grosicki, 2020), an uncommonly long HIFT workout. Though analyzed alongside several physiological measures of strength, power, and aerobic endurance, body fat percentage was the only significant correlate of overall time, explaining ∼51% of variance. Nevertheless, the lack of methodological consistency across studies limits the ability to make generalized conclusions about the role of body composition on HIFT performance.

Sample characteristics, particularly about experience, also differed greatly across the four studies relating body composition to HIFT (Butcher et al., 2015; Carreker and Grosicki, 2020; Mangine et al., 2020; Zeitz et al., 2020). Participants ranged from having no experience (Zeitz et al., 2020), 6–24 months of experience (Carreker and Grosicki, 2020; Mangine et al., 2020), or they had several years of HIFT experience, including regional and international competition experience (Butcher et al., 2015; Mangine et al., 2020). More time spent participating in a sport provides an athlete with more opportunities to develop and refine relevant skills and strategies that may help them overcome a physically or physiologically superior opponent. Still, HIFT experience is yet another documented predictor of performance that has received limited attention (Bellar et al., 2015; Mangine et al., 2020; Mangine and McDougle, 2022). This is interesting because one of the first HIFT prediction studies found years of experience to be the best predictor for two novel workouts; it was a better predictor than age, aerobic capacity, and anaerobic power (Bellar et al., 2015). However, that finding was slightly misleading because athletes with several, high-level HIFT competition experiences were being compared to those with no HIFT experience. It remained unclear whether experience with the traditional training modalities that comprise HIFT (e.g., resistance training, gymnastics, endurance training), years of HIFT participation, or the participants’ competition experiences were driving those relationships. This question was partially addressed in a later study that found that HIFT competition experience (and ranking) was more influential on performance than years of resistance training or HIFT experience (Mangine et al., 2020). Competition experience was then further evaluated and found to differentially influence performance and this was based on whether the athlete possessed experience as an individual or team competitor at open/local, regional, and international events (Mangine and McDougle, 2022). Though competition performance would seem to be the most standard and reliable metric for quantifying skill in HIFT, not all studies have recruited participants with such experience. Thus, an alternative could be to use the individual’s performance in benchmark workouts as a descriptor and/or inclusionary criteria. These are familiar, standardized workouts that more frequently appear in programming and are often tracked on HIFT-related message boards and social media websites (e.g., CrossFit, 2022). Despite being limited by the self-reported nature, normative values have been established from leaderboard data for five of the most common benchmark workouts appearing in HIFT (Mangine et al., 2018).

Unlike most physiological and performance measures, the relevance of body composition to performance is less obvious. Greater non-bone lean mass (NBLM), bone mineral content (BMC), and bone mineral density (BMD) are characteristics that support greater force and power expression (Lieber and Fridén, 2000; Schipilow et al., 2013; Stock et al., 2017). Conversely, athletes with less fat-mass (FM) and a lower percentage of body fat (PBF) may sustain effort better than individuals with greater non-functional mass due to a reduced relative workload, and potentially, a more efficient thermoregulatory system (O’Connor and Slater, 2011; Dervis et al., 2016). Still, any advantage awarded by superior body composition would seem to be modulated by the individual’s overall skill in that sport. Greater familiarity with a movement pattern leads to greater and more efficient muscle activation and a reduced relative workload (Krakauer et al., 2019). Likewise, strategies learned from participating in a sport may limit the occurrence of inefficient and unnecessary actions (Brenner, 2016; Myer et al., 2016). These advantages would collectively be useful in HIFT competition, which may require sustained activity, precise weightlifting and gymnastic movement execution, strength and power to lift heavier loads, or a combination of all three. How experience or sports skill may affect these needs remains unexplored. Therefore, the purpose of this study was to begin examining the influence of competition class and skill on the relationships between body composition and HIFT performance, where skill was defined by their performance in one of the most popular benchmark workouts (i.e., “Fran”). It was hypothesized that differences in all measures would exist between competition classes and percentile ranks. However, regardless of competition class and percentile rank, the relationships between measures of body composition and performance would be the same.

## Materials and Methods

### Study Design

To examine differences in and relationships between body composition and HIFT performance across sex, skill level, and competition class, recreationally active adults with at least 6 months of HIFT experience were recruited for this study. During enrollment, participants were asked to provide their personal best time-to-completion for the benchmark workout “Fran.” This workout was selected because of its status as a benchmark workout that users may upload scores for on the most popular HIFT leaderboard (CrossFit, 2022). Additionally, its expected duration (approximately 2–9 min) consistently appears in HIFT (Feito et al., 2018b; Mangine et al., 2018) and unlike longer duration workouts appearing on leaderboards, its execution is more easily standardized across training facilities. Participants were grouped according to their sex- and age-determined competition class and by their within-class percentile rank for “Fran.” Published normative values by Mangine et al. (2018) were used to appropriately place men (<35 years), women (<35 years), master’s men (≥35 years), and master’s women (≥35 years) into their respective interquartile range (i.e., ≤25th percentile, 25–75th percentiles, or ≥75th percentile). Following enrollment, participants were then scheduled to complete all body composition assessments via dual-energy x-ray absorptiometry (DXA). Comparisons were initially made between competition classes and percentile ranks for all body composition variables. Then, relationships between body composition variables and “Fran” performance were assessed for the entire sample, each competition class, and percentile rank grouping.

### Participants

Following a description of all study procedures, a convenience sample of ninety-five adults [31.0 ± 6.8 years (19–56 years), 173.0 ± 8.7 cm (156.2–193.0 cm), 77.4 ± 12.0 kg (51.7–106.1 kg) who possessed an average “Fran” time of 5.3 ± 2.6 min (2.1–18.1 min) provided his or her written informed consent to participate in this study. Based on previously reported differences among competition classes (Mangine et al., 2018), G*Power (v. 3.1.9.7, Heinrich-Heine-Universität, Düsseldorf, Germany) determined that a minimum of 44 participants were needed to sufficiently observe differences between competition groups (Effect size of *f* = 0.68, *α* = 0.05, *β* = 0.95). All participants had been regularly (≥2 sessions per week) and currently participating in HIFT for at least 6 months and were free of any injury or health condition (i.e., pregnancy, cardiovascular, pulmonary, metabolic disease, or orthopedic) known to impact physical activity, as determined by health and physical activity questionnaire. The University’s Institutional Review Board approved all testing protocols and procedures for this study.

### Workout Performance

All participants provided their personal best score (i.e., time to completion) for the benchmark workout “Fran.” Briefly, “Fran” is a 3-round circuit of thrusters (i.e., barbell front squat into an overhead press) and pull-ups (Mangine et al., 2018; CrossFit, 2022). For each round the thruster load remains the same [Men: 95 lbs. (43.1 kg); Women: 65 lbs. (29.5 kg)] but repetitions for each exercise descend from 21 repetitions (round 1) to 15 repetitions (round 2) to 9 repetitions (round 3). Each set of thrusters begins with the loaded barbell on the floor. The athlete must pick up the barbell into the front rack position and descend to a full squat. The crease of the hip must clearly pass below the top of the knees in this position. The athlete must return to the starting position and immediately progress into an overhead press. A repetition is considered complete when the knees, hips, and arms are at full extension with the barbell overhead. For pull-ups, each repetition begins with the athlete hanging from a standard pull-up bar with their arms extended and feet off the ground. Athletes must pull themselves vertically so that their chin breaks the horizontal plane of the bar before returning to the start position. Pull-ups may be performed using strict control or with a “kipping” or “butterfly” technique, so long as the arms return to full extension at the bottom of each repetition. Repetitions are discounted and must immediately be repeated before progressing through the remaining workload if technical standards are not met. All participants completed the workout at their normal training facility under the supervision of a Level 1 certified coach prior to enrollment in this study.

### Body Composition Assessment

The Participants arrived at the Exercise Physiology Laboratory after having fasted for 4 h and having avoided caffeine and vigorous exercise for at least 12 h to complete body composition assessments. Initially, anthropometric measures were collected using an electronic scale (Tanita WB 3000, Arlington Heights, IL) to measure height (±0.1 cm) and body mass (±0.1 kg), which were then used to calculate body mass index [BMI; body mass divided by height (in m) squared]. Anthropometric measures were completed with participants standing barefoot, feet together, on the scale while wearing athletic clothing. Subsequently, participants were further assessed by DXA (Lunar iDXA, Lunar Corporation, Madison, WI) performed by the same researcher using standardized positioning procedures. Participants were asked to remove any metal or jewelry prior to laying supine on the DXA table for an entire body scan in “standard” mode using the supplied algorithms. Quality assurance was assessed by daily calibrations performed prior to all scans using a calibration block provided by the manufacturer. In addition to total PBF (±0.1%), BMC (±0.01 kg), BMD (±0.01 g cm^−2^), fat mass (FM; ±0.1 kg), and NBLM (±0.1 kg), gynoid and android PBF (±0.1%) were obtained using manufacturer algorithms and used for statistical analyses. NBLM values were used to calculate fat-free mass index (FFMI; NBLM + BMC divided by height [in m] squared) (VanItallie et al., 1990).

### Statistical Analysis

The Shapiro-Wilks test indicated that most variables were not normally distributed. Therefore, data was logarithmically transformed to satisfy this assumption prior to assessing differences and relationships. Separate two-way (Competition class x Percentile Rank) analyses of variance (ANOVA) were conducted on all transformed measures of body composition and “Fran” time. All significant main effects and interactions were further assessed using the Tukey’s Honest Significant Difference test. All between group differences were also evaluated using effect sizes (η^2^_P_: Partial eta squared) at the following levels: small effect (0.01–0.058), medium effect (0.059–0.137) and large effect (>0.138) (Cohen, 1988). Spearman’s bivariate and partial correlations were performed between “Fran” time and all body composition variables. The strength of observed relationships were interpreted using the following criteria: Trivial (<0.10), small (0.10–0.29), moderate (0.30–0.49), high (0.50–0.69), very high (0.70–0.90), or practically perfect (>0.90) (Hopkins et al., 2009). All statistical analyses were performed using JASP 0.14.1.0 (Amsterdam, Netherlands) with a criterion alpha set at *p* ≤ 0.05. All data is presented, untransformed, as mean ± SD.

## Results

The results of each ANOVA are presented in Table 1. No significant interactions between competition class and percentile rank were noted for any variable.

**TABLE 1:** Main effects and interactions between competition classes and percentile ranks.

	Competition class			Percentile rank			Interaction		
	F	p	η^2^_p_	F	p	η^2^_p_	F	p	η^2^_p_
Height	14.7	<0.001	0.35	1.0	0.359	0.02	0.2	0.969	0.02
BMI	13.7	<0.001	0.33	0.1	0.951	0.00	1.4	0.243	0.09
FFMI	33.1	<0.001	0.55	7.2	<0.001	0.15	0.9	0.502	0.06
Body mass									
Total mass	29.8	<0.001	0.52	0.7	0.518	0.02	0.7	0.675	0.05
Fat mass	0.9	0.425	0.03	7.9	<0.001	0.16	1.3	0.271	0.09
Non-bone lean mass	50.1	<0.001	0.64	7.7	<0.001	0.16	0.4	0.859	0.03
*Percentage fat*									
Android	0.5	0.672	0.02	11.3	<0.001	0.22	0.8	0.576	0.05
Gynoid	22.5	<0.001	0.45	13.3	<0.001	0.24	1.5	0.186	0.10
Total body	10.8	<0.001	0.28	14.0	<0.001	0.25	1.0	0.457	0.07
*Bone mineral*									
Content	18.8	<0.001	0.41	3.0	0.057	0.07	1.2	0.306	0.08
Density	8.5	<0.001	0.24	1.4	0.251	0.03	1.1	0.397	0.07
Fran performance									
Time	13.6	<0.001	0.33	127.8	<0.001	0.76	0.7	0.660	0.05
Percentile rank	0.2	0.914	0.01	146.0	<0.001	0.78	0.4	0.906	0.03

Main effects for competition class were observed for all variables except fat mass and the percentage of android fat. Post hoc analysis indicated that men and master’s men possessed greater height (mean difference = 9.0–13.9 cm, *p* ≤ 0.008), total body mass (mean difference = 15.6–21.7 kg, *p* < 0.001), NBLM (mean difference = 16.8–23.3 kg, *p* < 0.001), FFMI (mean difference = 3.3–5.3 kg m^−2^, *p* < 0.001), and BMC (mean difference = 0.66–0.96 kg, *p* < 0.001), as well as lower percent total body fat (mean difference = 5.2–8.1%, *p* ≤ 0.006) and percentage gynoid fat (mean difference = 8.5–13.3%, *p* < 0.001), than women and master’s women. Men also possessed a higher BMI (mean difference = 2.6–4.4 kg m^−2^, *p* < 0.001) and greater BMD (mean difference = 0.11–0.20 g cm^−2^, *p* ≤ 0.012) than women and master’s women, whereas BMI (mean difference = 3.2 kg m^−2^, *p* = 0.003) and BMD (mean difference = 0.18 g cm^−2^, *p* = 0.004) were only greater in master’s men compared to master’s women. Further, although men and master’s men completed “Fran” faster than their female counterparts (mean difference = 51–160 s, *p* ≤ 0.009), no differences in percentile rank were seen. No other differences were found between competition classes. Significant differences between competition classes are presented in Table 2.

**TABLE 2:** Significant differences between competition classes, regardless of percentile rank [mean ± SD (range)].

	Men		Women		M. Men		M. Women	
	n = 42		n = 30		n = 15		n = 8	
Height (cm)	176 ± 7	(160–193)	167 ± 6	(156–184)^†,‡^	181 ± 7	(167–192)	166 ± 5	(157–172)^†,‡^
BMI (kg m^−2^)	27.3 ± 2	(20.4–30.5)	24.2 ± 1.8	(20.2–29.1)^†^	26.1 ± 2.3	(21.9–30.3)	23 ± 3.4	(20.2–28.4)^†,‡^
FFMI (kg m^−2^)	23.7 ± 2.2	(18.6–27.2)	19.5 ± 1.8	(15.6–24.1)^†,‡^	22.9 ± 2.1	(19.1–26.0)	18.4 ± 1.6	(16.2–20.4)^†,‡^
Body mass (kg)								
Total mass	84.4 ± 8.9	(66.7–106.1)	67.3 ± 7.5	(51.7–90.8)^†,‡^	85.4 ± 7.3	(67.8–95.2)	63.5 ± 7.9	(55.2–78.6)^†,‡^
Fat mass	14.4 ± 5	(7.2–29.6)	15.6 ± 4.8	(8.5–29.5)	14 ± 4.6	(7.2–26)	15.2 ± 5.9	(9.7–25.2)
Non-bone lean mass	70 ± 8.4	(52.4–85.8)	51.7 ± 7.2	(42.2–78.1)^†,‡^	71.4 ± 7	(56.4–78.6)	48.4 ± 4.6	(41.7–55.2)^†,‡^
Percentage fat (%)								
Android	18.7 ± 8.7	(8–43)	19.7 ± 8.5	(8.7–43.3)	17.4 ± 6.8	(8.6–29.2)	19.5 ± 8.4	(10.4–31.3)
Gynoid	17.7 ± 5.7	(8.7–32.4)	27 ± 6.5	(13.2–44.8)^†,‡^	16 ± 3.4	(9.2–20.6)	29.2 ± 7.7	(20.9–42.4)^†,‡^
Total body	17.8 ± 5.4	(10.1–30.1)	23.4 ± 6	(13.3–40.9)^†,‡^	16.5 ± 3.7	(10.3–22.9)	24.4 ± 6.8	(17.2–35.1)^†,‡^
Bone mineral								
Content (kg)	3.47 ± 0.53	(2.3–4.48)	2.71 ± 0.39	(2.13–3.74)^†,‡^	3.54 ± 0.41	(2.73–4.31)	2.58 ± 0.38	(1.93–3.11)^†,‡^
Density (g cm^−2^)	1.39 ± 0.13	(1.16–1.71)	1.26 ± 0.1	(1.09–1.41)^†^	1.37 ± 0.12	(1.19–1.6)	1.19 ± 0.15	(0.95–1.4)^†,‡^
Fran performance								
Time (sec)	279 ± 128	(125–566)	337 ± 127	(155–721) ^†,‡^	309 ± 149	(140–660)	460 ± 271	(254–1085)^†,‡^
Percentile rank (%)	53.2 ± 32.7	(11.9–99.9)	50.2 ± 22.9	(16.5–98.4)	59.6 ± 32.4	(15–100)	56.7 ± 30.7	(20.4–100)

†, significantly (*p* < 0.05) different than men.

‡, significantly (*p* < 0.05) different than masters men.

**FIGURE 1:**
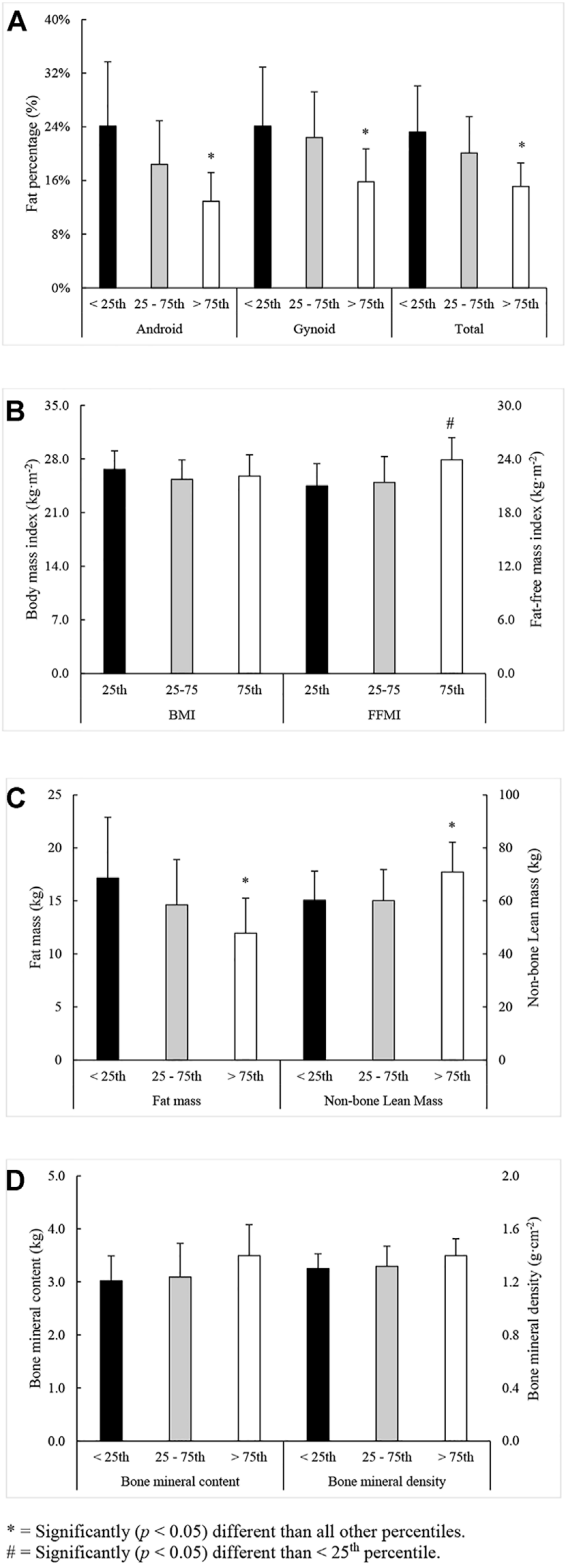
Significant differences between percentiles in measures of **(A)** body fat percentage, **(B)** body mass and fat-free mass index, **(C)** fat and non-bone lean mass, and **(D)** skeletal mass characteristics (mean ± SD). * = Significantly (*p* < 0.05) different than all other percentiles. # = Significantly (*p* < 0.05) different than <25th percentile.

Significant main effects for percentile rank were observed for “Fran” performance, body fat percentage (android, gynoid, and total), FM, FFMI, and NBLM. Regardless of competition class, individuals from >75th percentile (“Fran” time = 167 ± 32 s, 81 ± 3 percentile rank, *p* < 0.001) completed “Fran” faster than those within the 25th–75th percentiles (“Fran” time = 283 ± 74 s, 53 ± 16 percentile rank) and below (“Fran” time = 485 ± 144 s, 10 ± 9 percentile rank). Those ranking between the 25th–75th percentiles were also faster (*p* < 0.001) than those ranking below. Those from >75th percentile possessed lower fat percentage (android, gynoid, and total), less FM, and more NBLM than all other percentiles. Those ranking above the 75th percentile also possessed a greater FFMI than those below the 25th percentile. No other differences were seen between percentiles. The differences between percentiles for measures of body composition are illustrated in Figure 1.

Bivariate and partial correlations between “Fran” time and body composition measures are presented in Table 3. Significant (*p* < 0.05) bivariate and partial (controlling for competition class) correlations were found between “Fran” time and all measures of body composition, with differences in each’s ability to explain variance ranging between 5.0% and 34.6%. These relationships were altered when the analysis was repeated after splitting the sample by competition class. In men, all body composition measures except for height were related to “Fran” time, whereas significant (*p* < 0.05) relationships were limited to percent fat (android, gynoid, total), FM, NBLM, and FFMI in women. Within the master’s class, fewer relationships were seen. Percent android and total fat, as well as FFMI, were the only measures related to “Fran” time in master’s men, and no significant relationships were seen in master’s women.

**TABLE 3:** Significant relationships between “Fran” time and measures of body composition.

	Bivariate	Partial Correlation		Men	Women	M. Men	M. Women
				>25th percentile	25–75th percentile	>75th percentile	-
Height (cm)	-0.26*	Competition class	-0.22*	-0.19	-0.24	0.20	-0.36
		Percentile rank	-0.31*	-0.12	-0.38*	-0.45*	-
BMI (kg m^−2^)	-0.31*	Competition class	-0.25*	-0.31*	0.11	-0.47	-0.24
		Percentile rank	-0.48*	-0.36	-0.49*	-0.53*	-
FFMI (kg m^−2^)	-0.58*	Competition class	-0.55*	-0.65*	-0.40*	-0.59*	-0.38
		Percentile rank	-0.60*	-0.63*	-0.59*	-0.51*	-
Body mass (kg)							
Total	-0.35*	Competition class	-0.30*	-0.40*	0.06	-0.17	-0.17
		Percentile rank	-0.47*	-0.32	-0.49*	-0.57*	-
Fat mass	0.39*	Competition class	0.39*	0.34*	0.55*	0.41	0.29
		Percentile rank	0.12	0.11	0.25	-0.03	-
Non-bone lean mass	-0.50*	Competition class	-0.46*	-0.57*	-0.41*	-0.44	-0.62
		Percentile rank	-0.52*	-0.37*	-0.54*	-0.63*	-
Percentage fat (%)							
Android	0.50*	Competition class	0.51*	0.53*	0.53*	0.53*	0.11
		Percentile rank	0.16	0.01	0.33*	-0.02	-
Gynoid	0.53*	Competition class	0.50*	0.48*	0.68*	0.28	0.43
		Percentile rank	0.48*	0.32	0.60*	0.37	-
Total	0.59*	Competition class	0.57*	0.53*	0.65*	0.53*	0.43
		Percentile rank	0.41*	0.23	0.53*	0.27	-
Bone mineral							
Content (kg)	-0.44*	Competition class	-0.40*	-0.52*	-0.23	-0.08	-0.24
		Percentile rank	-0.46*	-0.24	-0.52*	-0.62*	-
Density (g cm^−2^)	-0.41*	Competition class	-0.37*	-0.47*	-0.12	-0.06	-0.24
		Percentile rank	-0.41*	-0.24	-0.41*	-0.59*	-

*, significant *(p <* 0.05) relationship between variables.

**FIGURE 2:**
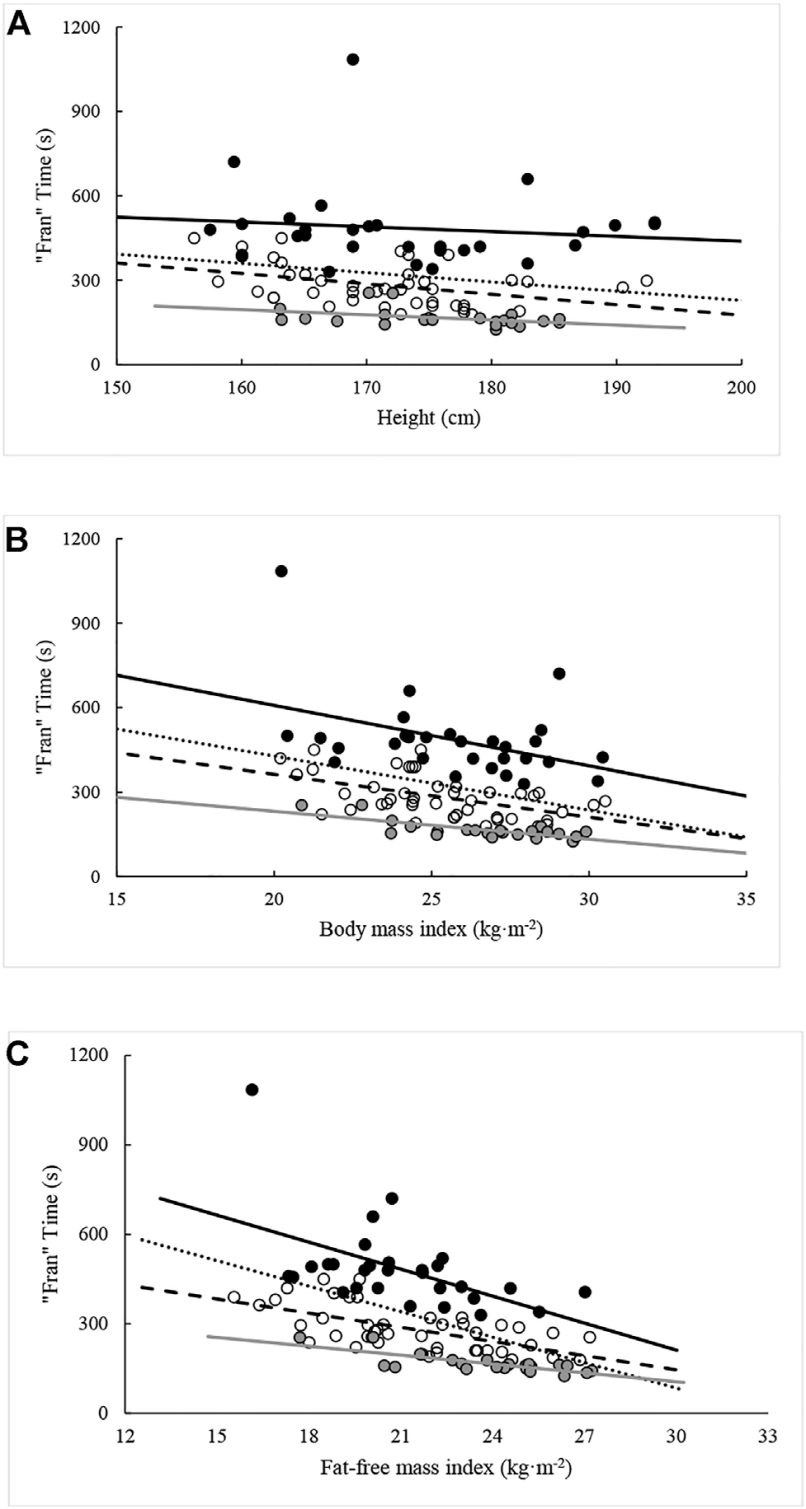
Relationships between “Fran” time and **(A)** height, **(B)** BMI, and **(C)** FFMI across percentile ranks. Note: Dotted regression line (*n* = 95), black spheres and regression line (*n* = 29, <25th percentile), open spheres and dashed regression line (*n* = 44, 25–75th percentiles), and grey spheres and regression line (*n* = 22, >75th percentile).

**FIGURE 3:**
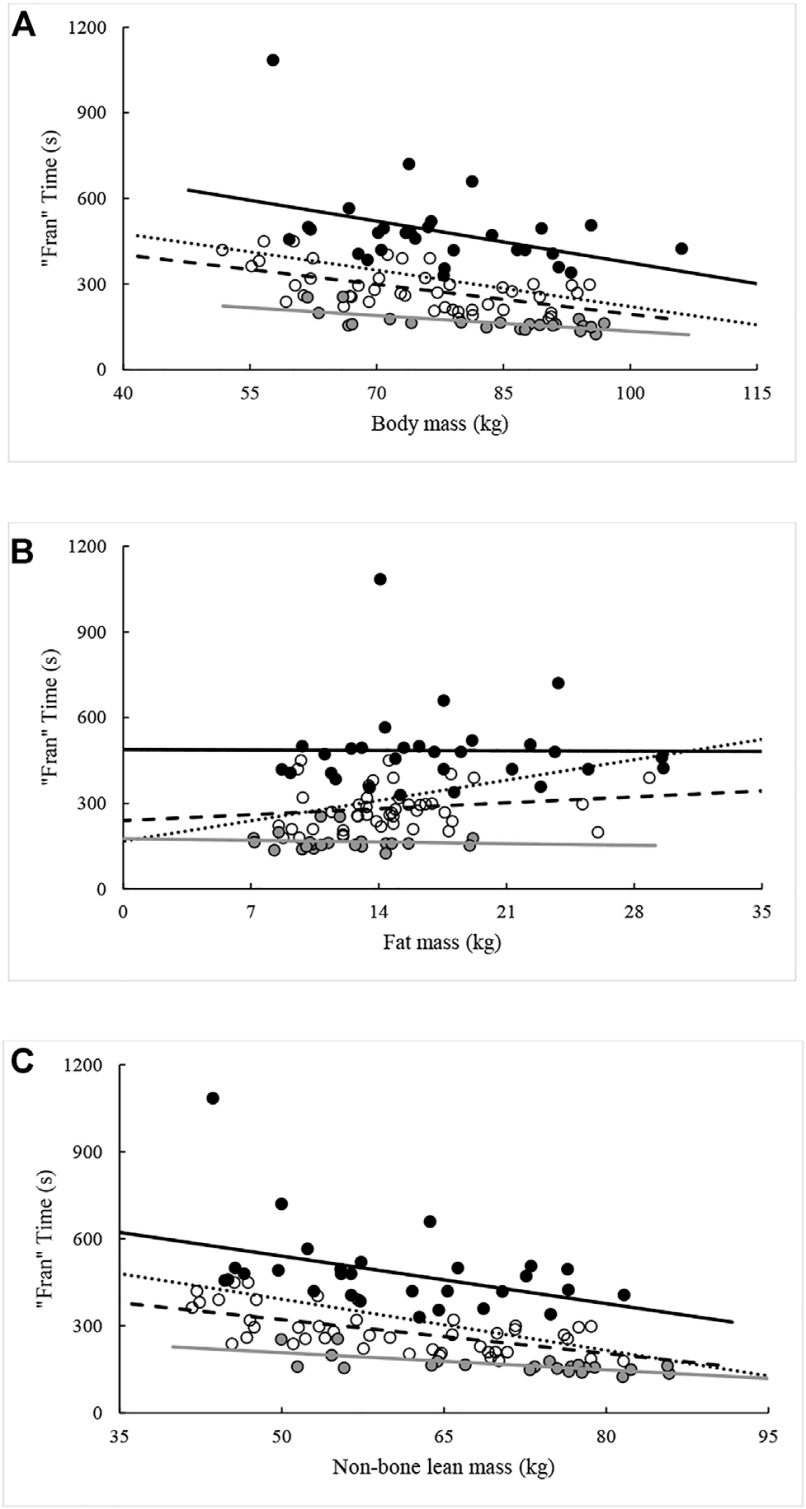
Relationships between “Fran” time and **(A)** body mass, **(B)** fat mass, and **(C)** non-bone lean mass across percentile ranks. Note: Dotted regression line (*n* = 95), black spheres and regression line (*n* = 29, <25th percentile), open spheres and dashed regression line (*n* = 44, 25–75th percentiles), and grey spheres and regression line (*n* = 22, >75th percentile).

**FIGURE 4:**
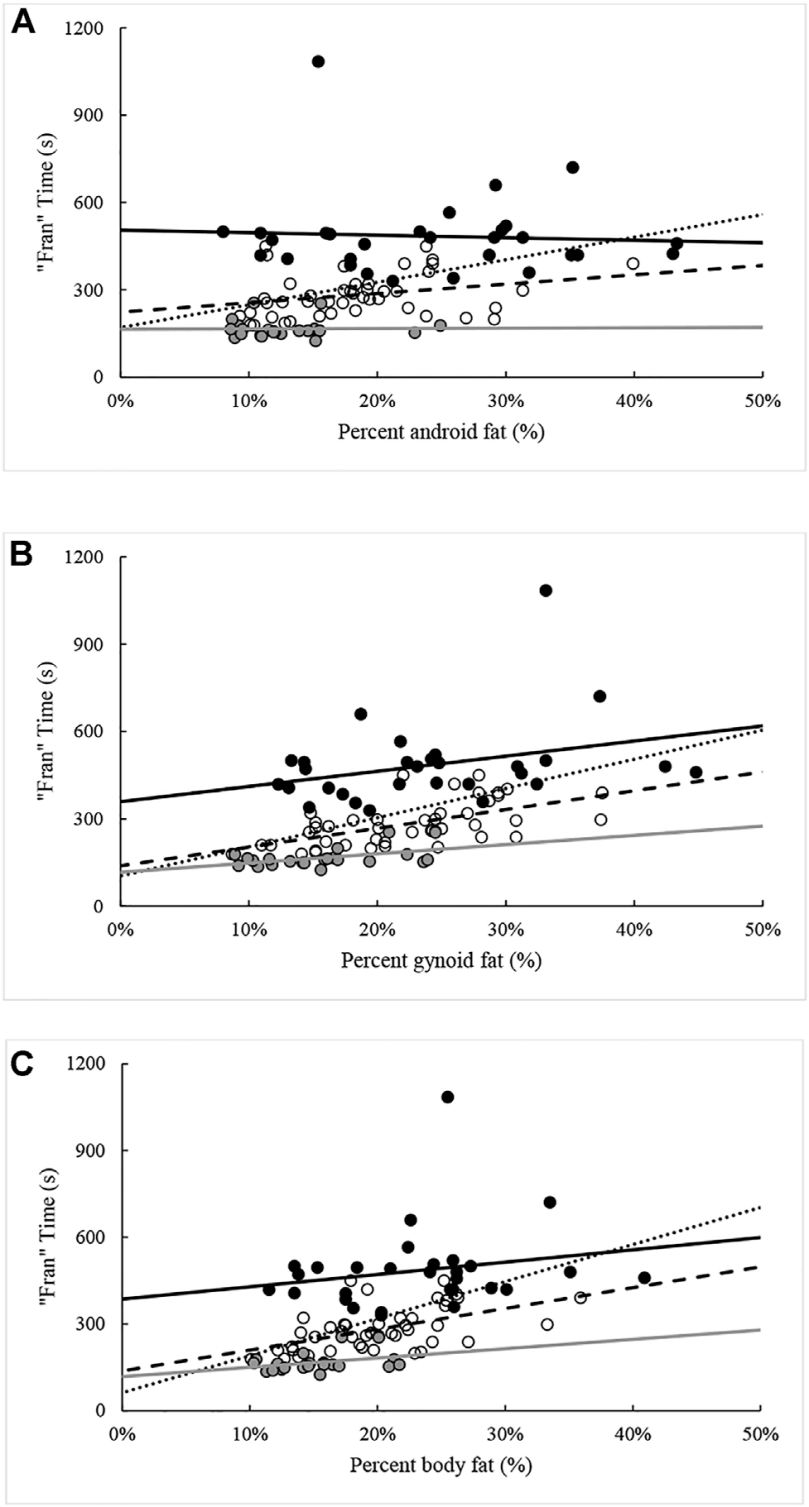
Relationships between “Fran” time and percentage **(A)** android fat, **(B)** gynoid fat, and **(C)** total fat across percentile ranks. Note: Dotted regression line (*n* = 95), black spheres and regression line (*n* = 29, <25th percentile), open spheres and dashed regression line (*n* = 44, 25–75th percentiles), and grey spheres and regression line (*n* = 22, >75th percentile).

**FIGURE 5:**
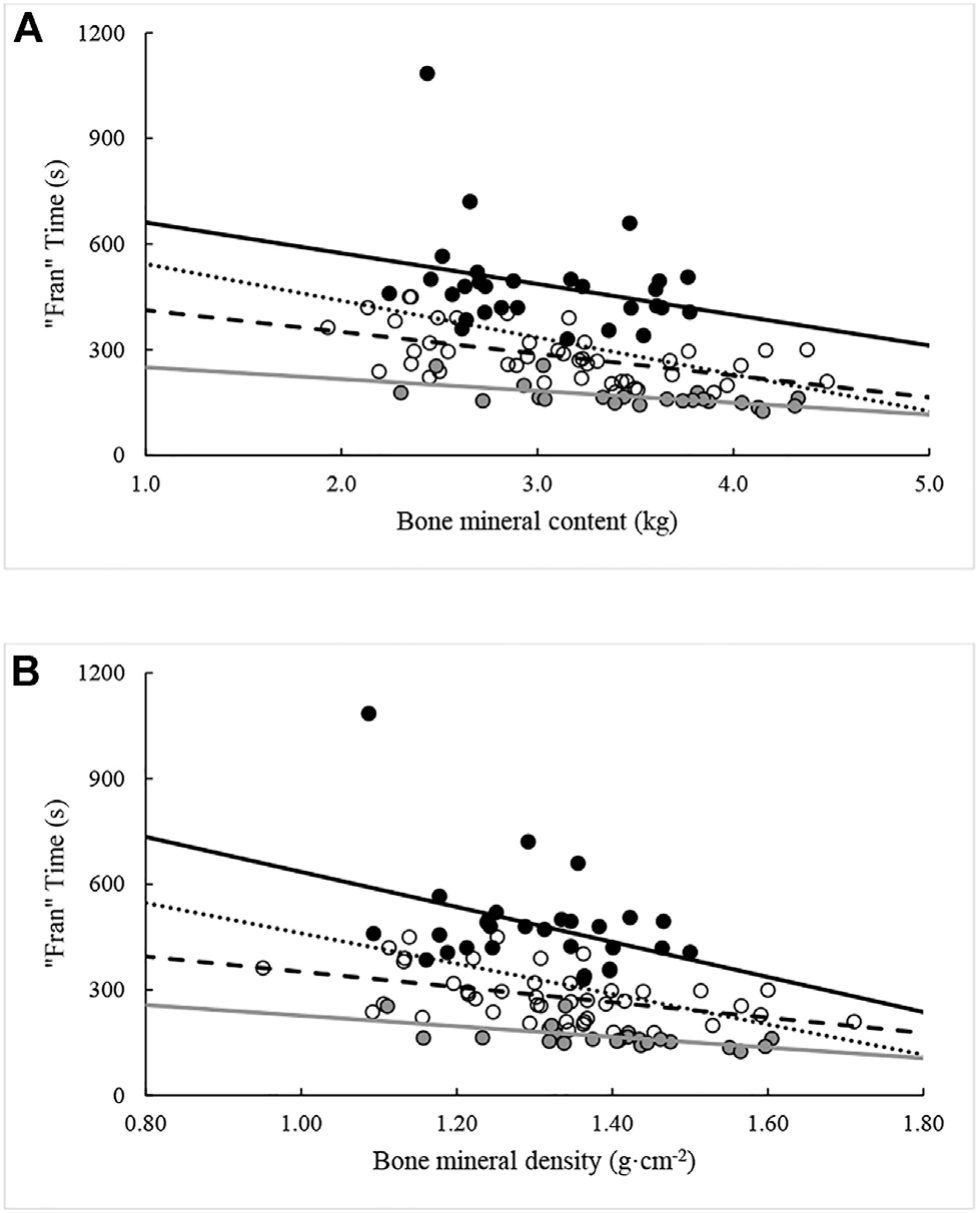
Relationships between “Fran” time and bone mineral **(A)** contend and **(B)** density across percentile ranks. Note: Dotted regression line (*n* = 95), black spheres and regression line (*n* = 29, <25th percentile), open spheres and dashed regression line (*n* = 44, 25–75th percentiles), and grey spheres and regression line (*n* = 22, >75th percentile).

Except for FM and percent android fat, all measures were again significantly (*p* < 0.05) related to “Fran” time when controlling for the influence of percentile rank. The ability of each variable in explaining variance in “Fran” time ranged between 9.6% and 36.0%. When the analysis was repeated with the sample split by percentile rank groupings, different combinations of significant relationships were seen within each grouping. All variables except FM were related to “Fran” time for participants ranking between the 25th and 75th percentiles. Likewise, all body composition variables, except those relating to fat distribution [i.e., FM and percent fat (android, gynoid, total)], were related to “Fran” time in >75th percentile. In contrast, only NBLM and FFMI were related to “Fran” time in participants from <25th percentile. The effects of percentile rank on relationships between “Fran” time and measures of body composition are illustrated in Figures 2–5.

## Discussion

This study aimed to assess the influence of competition class and percentile rank on relationships between body composition and HIFT performance using the benchmark workout “Fran.” Though nearly a handful of studies have reported relationships between various measures of body composition and one or more HIFT workouts (Butcher et al., 2015; Carreker and Grosicki, 2020; Mangine et al., 2020; Zeitz et al., 2020), any consensus is clouded by several methodological differences existing amongst these studies. One limited relationships to simply height and body mass (Butcher et al., 2015), two related performance to DXA-derived PBF (Carreker and Grosicki, 2020; Zeitz et al., 2020), and only one examined multiple body composition compartments (Mangine et al., 2020). The strength of their reported relationships, including whether they were significant, also depended on the specific workout being used to define HIFT performance. Across all studies (Butcher et al., 2015; Carreker and Grosicki, 2020; Mangine et al., 2020; Zeitz et al., 2020), the included HIFT workouts only appeared once except for the CrossFit® total (i.e., the sum of 1-RM deadlift, back squat, and overhead press) (Butcher et al., 2015; Zeitz et al., 2020). More importantly, and relevant to this study, none of the studies considered the influence of competition class and percentile rank on these relationships. Here, we built upon past work (Butcher et al., 2015; Zeitz et al., 2020) by reexamining “Fran” with a much larger sample, a more comprehensive usage of DXA, and by distinguishing relationships by competition class and percentile rank.

Men generally possessed more lean mass and less fat mass, and their “Fran” times were faster than those seen in women, but no differences were seen across age groups. In healthy, athletic populations, men are well-known to possess more muscle and less fat than women, and these differences may help explain why men typically perform better (Tseng et al., 2014; Jagim et al., 2019; Huebner and Perperoglou, 2020). HIFT programming tries to account for the known physiological differences between men and women by scaling workouts. For “Fran,” this is accomplished by prescribing different intensity loads for thrusters [i.e., 95 lbs. (43.1 kg) for men and 65 lbs. (29.5 kg) for women] but nothing is altered for pull-ups (Feito et al., 2018b; Mangine et al., 2018). The rationale for why pull-up prescription is the same for men and women is not clear. A recent study reported a strong correlation between “Fran” time and maximum strict pull-ups (*r* = −0.598) (Leitão et al., 2021). Although stronger relationships were seen with thruster strength and endurance (*r* = −0.608 to −0.822), upper-body strength endurance is clearly important. Indeed, an individual must have the capacity to complete a total of 45 pull-ups to finish “Fran.” While there is evidence of women being more resistant to upper-body fatigue than men (Hunter, 2016), they have historically had more difficulty performing multiple, consecutive pull-ups (Flanagan et al., 2003). This is likely because the intensity of pull-ups is defined by the individual’s body mass. Body mass and lean mass have been previously associated (negatively) with pull-up performance (Johnson et al., 2009; Sánchez Moreno et al., 2016). On average, body mass and composition, particularly when considering its distribution, are not the same between men and women (Tseng et al., 2014; Jagim et al., 2019; Huebner and Perperoglou, 2020). Being heavier, men should have a more difficult time performing consecutive pull-ups. However, because men typically possess more upper-body lean mass, they have more relevant, functional mass to devote to pull-ups. Even when normalizing for body mass and lean mass (i.e., per kg), greater pull-up strength has been documented in men (Johnson et al., 2009). Women only equaled men when the load was perfectly equated (i.e., as a covariate) (Johnson et al., 2009), an inappropriate statistical procedure when natural differences between groups prevent random assignment (Weir and Vincent, 2005). Women might overcome this natural disadvantage by employing a “kipping” or “butterfly” technique and redirecting some of the work to the lower-body (Williamson and Price, 2021), but since both sexes are permitted this option, the gap between sexes remains. This is supported by “Fran” time generally being related positively to fat mass and PBF, and negatively to NBLM and FFMI.

The lack of differences between age groups, as well as the fewer significant relationships seen between body composition and “Fran time” in the master’s class, are most likely the consequence of reduced statistical power. There were nearly three times as many younger participants as those who were older than age 35 years. While this may be viewed as a limitation to this study, and potential source of type II error, these numbers are consistent with the ratios seen between master’s and younger athletes in Open and international competition (Leaderboard, 2021). Nevertheless, an equal or greater (but non-significant) correlation coefficient was seen in master’s participants for approximately one-third of the variables found to be significantly related to “Fran” time in younger participants. Additionally, the master’s class begins at age 35 years, and the oldest participant in the present study was 56 years old. Despite this 20-year range, appreciable changes to physiology, particularly in physically-active, resistance-trained adults, are less common than they are with similarly aged, sedentary adults (McGregor et al., 2014; Larsson et al., 2019). Since no significant differences were found between younger and older participants, theoretically, the relationships between body composition and “Fran” time should have been the same. Thus, for the time being, these findings should be viewed as preliminary.

Participants from >75th percentile possessed less fat mass and more NBLM than all other participants. Meanwhile, no differences were seen among the lower percentile groups or with any measure of bone health. The size, architecture, and quality of skeletal muscle reflect its ability to produce force (Lieber and Fridén, 2000; Stock et al., 2017). The mass and density of bone are also thought to contribute to force production by providing a stable structure through which force may transfer and elicit human movement. However, there is less evidence available documenting an advantage from exercise-induced gains in bone size (Schipilow et al., 2013) and adaptations require longer training periods (6–8 months) (Kohrt et al., 2004). In the present study, NBLM and FFMI were related to “Fran” time for all percentile ranks, whereas BMC and BMD were related to performance in everyone except the lowest percentile. It is possible that lower-ranked individuals must sufficiently develop a variety of physiological traits and/or sport-specific skills before bone mass becomes a relevant factor. Regardless, these findings provide support for previous reports of “Fran” time being highly correlated to performance measures of muscular strength (Butcher et al., 2015; Zeitz et al., 2020; Leitão et al., 2021) and endurance (Leitão et al., 2021).

Interestingly, PBF measures were only relevant to those ranking within the interquartile range (i.e., 25th–75th percentile). A leaner individual might use less energy when performing repeated movements at a given intensity, and assuming proper hydration and ventilation, thermoregulate better than someone with a higher body fat percentage during exercise (O’Connor and Slater, 2011; Dervis et al., 2016). Together, these could prolong the onset of fatigue and better facilitate sustained movement during extended-duration exercise. However, the relevance of this advantage to “Fran” is unclear. For most individuals, regardless of competition class, the average completion time for “Fran” ranges between 4 and 6 min (Mangine et al., 2018), which more closely resembles anaerobic effort than a long-duration aerobic event. Indeed, respiratory exchange ratio values have been reported to be greater than 1 (indicating anaerobic metabolism) for more than 75% of “Fran” (Fernandez-Fernandez et al., 2015), and the workout is also highly correlated (*r* = 0.673) with the 2K rowing time (Interquartile range = 7.3–7.7 min) (Leitão et al., 2021), another predominantly anaerobic event. For the lowest-ranking participants in this study, the need to improve lean mass appears to supersede all other needs (physiological and technical). Their average times ranged between 7.3 and 11.4 min, and up to 18.1 min. Within the context of this workout, being unable to lift the assigned thruster load for multiple repetitions, or perform pull-ups sequentially, would seem to be the most likely explanations. Meanwhile, the highest-ranked individuals, who also possessed the healthiest body composition, may have reached a point where continued focus on PBF reduction was either unnecessary or unhealthy. Instead, continuing to improve lean mass to further force production capabilities, and possibly perfecting technique may prove more beneficial. In contrast, though middle-ranked individuals may still benefit from improved lean mass, more rapid improvements in “Fran” might happen with a healthier PBF.

### Conclusion

The findings of this study indicate that the various sub-categories of body composition are all related to “Fran” performance, but their individual relevance is modulated by competition class and skill. Despite the compositional differences seen between men and women, relationships to performance were similar for each sex. The lack of age group differences within each sex, and significant relationships to performance in the master’s class, are contrary to this conclusion. However, this was likely because less participants qualified for the master’s class and thus, reduced statistical power. A more deliberate effort in recruiting sufficient participants within each competitive class will help to clarify this disagreement. Across percentile ranks, the higher-ranking participants (>75th percentile) possessed more NBLM and less body fat than all other participants, and those who possessed more lean mass (NBLM, FFMI, BMC, and BMD) performed better. Although middle- (25th–75th percentiles) and lower-ranking (<25th percentile) participants possessed similar body composition, the relationships of each sub-category to performance were different. Moderate to high correlations with “Fran” time were noted for all sub-categories (except FM) in middle-ranking participants, whereas NBLM was the only sub-category associated with performance in the lower-ranking participants. Including assessments of muscular strength in the thruster exercise and maximal pull-up repetitions (using all relevant styles) would have helped to better explain the practical importance of NBLM to performance. These findings are also limited to self-reported “Fran” times. Future studies may want to confirm our findings by directly testing “Fran” or expand on them by including a greater variety of benchmark workouts. Nevertheless, this appears to be the first study to examine the influence of competition class and percentile rank on relationships between any physiological measure and HIFT performance.

### Practical Applications

The findings of this study suggest that relationships between “Fran” time and body composition are important for both men and women. Striving for a healthy ratio of NBLM to fat mass appears to be related to a faster “Fran” time but men and women may accomplish this differently. In men, greater body mass and bone mineral content/density were relevant to performance, and these traits are typically enhanced when long-term training goals are to develop muscle size, strength, and power. In women, body and skeletal mass were not related to “Fran” time. Though the reasons for this are unknown, it may imply a greater reliance on movement efficiency rather than strength to complete workout tasks. Significant relationships were not found in master’s participants. Still, it may be prudent to assume that this was the consequence of reduced power. Master’s class adults should seek to model their training goals after their younger counterparts. When the analysis considered percentile rank, NBLM was related to performance in all participants, and the strength of this relationship increased in those who completed “Fran” in less time. By improving NBLM, strength is presumably increased, and this would reduce the relative intensity of the fixed loads prescribed for this workout. Meanwhile, attention to PBF and fat mass reduction only appears to be relevant for moderately ranked individuals. More skilled participants possessed the healthiest fat-to-lean mass ratio, and this seems to suggest that a threshold exists where continued focus on this goal has no additional benefit. In the lowest ranked participants, the only relationship observed was between NBLM and “Fran” time. This may reflect a need to improve strength, technique, pacing strategy, or possibly all three. Any concerted effort to reduce fat mass at this stage seems to be premature.

## Data Availability

The raw data supporting the conclusion of this article will be made available by the authors, without undue reservation.
